# Features of Mobile Health Apps for Tobacco Cessation That Appeal to Black Adults Who Use Tobacco Products: Focus Group Study

**DOI:** 10.2196/63340

**Published:** 2026-02-06

**Authors:** Sonia A Clark, Remi Philips, Christine E Kistler, Adam O Goldstein, Chineme Enyioha

**Affiliations:** 1 Lineberger Comprehensive Cancer Center, University of North Carolina at Chapel Hill, Chapel Hill, NC, United States; 2 Department of Family Medicine, University of North Carolina at Chapel Hill, 590 Manning Dr, Chapel Hill, NC, 27514, United States, 1 984-215-5048; 3 Division of Geriatrics, University of Pittsburgh School of Medicine, Pittsburgh, PA, United States

**Keywords:** mHealth, tobacco cessation, preferred features, mobile health app, representation, inclusivity, African American, Black adult tobacco users, mobile health

## Abstract

**Background:**

Mobile health (mHealth) interventions show promise in supporting tobacco cessation. However, Black adults who use tobacco products are not well represented in mHealth studies for tobacco cessation, and their preferred features of mHealth apps are not well known. Identifying types of mHealth app features for tobacco cessation preferred by Black adults is critical to developing a culturally adapted app, with increased uptake by the target population.

**Objective:**

The goal of this study was to explore culturally relevant preferences for features of smoking cessation mHealth apps among Black adults who use tobacco products.

**Methods:**

A comprehensive list of features of mHealth apps for tobacco cessation was developed based on previous research and a review of existing mHealth literature. Through a content analysis, this list was divided into subgroups and used to develop a focus group guide. We recruited participants from Instagram, a social media platform. Eligible focus group participants included people who reported current use of a tobacco product, identified as being African American or Black, were 21 years old or older, and had access to Wi-Fi or the internet. Participants had to indicate interest in the use of an mHealth app for tobacco cessation. Participants discussed their opinions about different app features, including what features they felt would increase the use of an app by Black adults. Recordings from the focus groups were transcribed and coded deductively and inductively. We conducted a thematic content analysis of the resulting transcripts.

**Results:**

Forty adults aged 21‐69 (mean 43, SD 13.6) years participated in 8 focus groups. Fifty-seven percent were female, and 88% endorsed current cigarette use. Four central themes that represented app features emerged. (1) Participants wanted representation and inclusivity through personalization and featuring people with similar lived experiences, including representative images and relevant health information. (2) Participants desired the app to feature a diversity of experiences such as testimonials from individuals from different backgrounds rather than solely focusing on racial identity or excessive targeting of the Black community. (3) Participants desired accountability through trusted connections with health care professionals and other support groups within the app, as well as app tracking capability. (4) Encouragement and motivation were more salient incentives than monetary rewards.

**Conclusions:**

Black adults who use tobacco products prefer a tobacco cessation app with features that are inclusive, relatable, supportive, and motivating. These findings can serve as the groundwork for the development of an mHealth app that will appeal to Black adults, potentially leading to increased app use, successful cessation, and health equity.

## Introduction

In the United States, certain racial minorities, including Black adults, face a disproportionate burden of negative health effects from tobacco use [1]. Compared to other racial and ethnic groups, Black adults who use tobacco products are more likely to experience adverse health issues because of their tobacco use, such as coronary heart disease, and have a higher morbidity and mortality rate from other tobacco-related health conditions [2,3]. For instance, compared with Whites, Black adults have a higher mortality rate from cancer (221 vs 186 per 100,000 population) [4]. Despite starting to smoke at an older age [5] and being more likely to make a quit attempt than any other racial group, Black adults who use tobacco products are less likely to successfully quit than other racial groups [6]. Black adults also have lower odds of both quitting and using Food and Drug Administration cessation medications, despite having greater odds of using behavioral counseling/self-help materials [7].

Novel interventions, such as mobile health (mHealth) interventions, show promise in supporting tobacco cessation for Black adults. mHealth apps have previously been successfully used with Black adults to help manage conditions such as diabetes and obesity [8,9]. Compared to White adults, Black adults are more likely to use apps to inform their medical decisions [10]. mHealth apps can also increase access for Black adults who use tobacco products by reducing barriers to receiving care, like cost and transportation logistics [11,12]. One study examining willingness to participate in mHealth app research for weight management among Black women found high receptivity, frequent smartphone usage, and existing use of smartphones to access information on health and wellness, suggesting mHealth apps could be used to empower and promote participation [13].

Although a promising route to increase tobacco cessation, mHealth apps should be culturally adapted to address unique factors that impact Black adults who use tobacco products. Black adults use menthol products and smoke cigars at higher rates [14], smoke fewer cigarettes per day [15], and may be more likely to use tobacco products as a result of stress related to racial harassment [16]. One study examining differences in addiction and quitting experiences concluded that these differences may suggest that Black adults who use tobacco products experience a different “quitting process” [15] as compared to White smokers. However, Black adults who use tobacco have not been well represented in prior mHealth studies for tobacco cessation, and their preferred features of mHealth apps are not well known. In addition, research has shown that tailoring interventions in ways that incorporate values and cultural experiences for patients, especially patients from minority backgrounds, can increase engagement with the intervention [17]. For example, visual representation of an individual’s culture in an intervention, delivery or promotion of an intervention by an individual from the target population, or the use of language that the target audience can relate to can enhance the appeal of that intervention [17,18]. A critical step to developing a tobacco cessation mHealth app that Black adults are receptive to and are likely to use is to identify desirable app features.

Furthermore, the extent to which preferred features satisfy key components of the self-determination theory is also important. According to the self-determination theory, autonomy, competence, and relatedness are key factors necessary for intrinsic motivation as well as maintenance of behavior change [19]. Autonomy, described as the intrinsic need to regulate one’s own behavior without the influence of external factors, has been found to be effective in tobacco cessation [20]. Competence, also described as the need for an individual to feel confident and capable as it relates to behavior change, can be supported by app features such as an app’s ability to provide information about tobacco cessation or provision of information about a user’s progress or behavior [21]. Relatedness is described as connecting with others and can be achieved by an app that allows for social support or interaction with others [21]. Each component of the self-determination theory plays an important role in cessation-related behavior [20,22]. The goal of this study was to identify features of mHealth apps for tobacco cessation that Black adults who use tobacco products would like to see in an app and what features will make the app more inclusive [19-22].

## Methods

### Study Design

Between June and July of 2023, we conducted 8 focus groups with 40 Black adults who use tobacco products. Participants were recruited using the social media platform Instagram. After completing a brief screener and obtaining consent during a brief screening call, eligible participants were purposively invited to participate in focus groups. Focus groups were stratified by age groups (21‐34, 35‐54, and ≥55 years old) to optimize representation.

### Participants

Eligible participants included Black adults who were 21 years old or older, who reported current use of one or more tobacco products in the past 30 days, spoke English, and were current US residents at the time of recruitment. Exclusion criteria included lack of access to the internet and lack of interest in the use of technology for smoking cessation.

### Focus Group Guide Development

To identify which features to discuss during focus groups, we developed a comprehensive list of features of mHealth apps for tobacco cessation using previous research and a review of existing mHealth literature [23-31]. The mHealth apps involved were designed based on behavior change techniques and theories, such as the principles of acceptance and commitment therapy [24,26], persuasive technology for behavior change [27], or followed the US clinical practice guidelines [28,31]. One of the apps was developed by the National Cancer Institute [28], and another was developed to target young adults who are motivated to quit [27]. We then divided this list into different domains to explore with participants. Initial mHealth app feature domains included content, user experience, personalization, tracking, privacy and security, connections or support, and inclusivity. Within each domain, several features were included to discuss with participants, with the primary focus being whether these features were inclusive and preferred by Black adults who use tobacco products. Using guidance from experts, the resulting domains and features were organized and distilled. The list and resulting domains were further refined through focus groups using the focus group guide.

### Focus Group Procedures

Using the list of domains and features of mHealth apps, the research team developed the focus group guide to address the research question: What features in mHealth apps for tobacco cessation are preferred by Black adults? The interview guide included questions and probes about participants’ experience with tobacco use and cessation, and experience with mHealth apps. Next, the guide listed each of the domains and features with a corresponding question about what features they preferred and a final question to get feedback about other design features or advice participants had. The semistructured focus groups were conducted using Zoom videoconferencing software to allow for participation from different locations within the United States, and each focus group lasted an hour [32].

### Analysis

The resulting focus group recordings were professionally transcribed, and transcripts were uploaded into Atlas.ti, a qualitative data analysis software (version 23). Using the focus group guide, an initial codebook was developed. The qualitative analysis team comprised 3 researchers (CE, SAC, and RP), who each had prior experience with qualitative coding and received formal qualitative and mixed methods training at the University of North Carolina at Chapel Hill. After familiarizing themselves with the codebook, 2 researchers (SAC and RP) independently coded a randomly selected transcript with the guidance of the senior researcher (CE). To establish the rigor of the analysis, we applied multiple methods to strengthen the credibility, dependability, and confirmability. First, we documented codebook revisions and applied analytic triangulation with multiple coders who reviewed transcripts independently and participated in consensus meetings to review discrepancies, add new codes, and refine definitions as needed. To assess interrater reliability, Krippendorff α was used, and the initial interrater reliability was calculated to be 0.769, indicating substantial agreement. After resolving coding conflicts, a different transcript was then coded, and “near perfect” agreement, 0.9, was reached. Upon reaching this threshold of high agreement, the team recoded the initial transcript and coded the remaining transcripts with the finalized codebook.

Furthermore, the focus groups were conducted with cultural sensitivity. For instance, the main facilitator of the focus groups was a member of the research team and is also a part of the Black community, as well as familiar with participants’ cultural values [32]. We did not conduct member check-in to reduce the burden on participants and concerns for confidentiality. However, the data were presented to the larger research team, and findings were discussed in detail [33].

Using thematic content analysis, the research team discussed and identified recurring themes and concepts across the transcripts [34]. The team determined that thematic saturation was achieved after the completion of 8 focus groups [35].

### Ethical Considerations

This study was approved by the institutional review board at the University of North Carolina at Chapel Hill (24‐0798). All study participants were informed about the study aims and significance, as well as the methods. Participants gave their informed consent, retained the rights to withdraw from the study at any time, and were informed of the confidentiality and anonymity of the data and the goal to publish findings from the study. All participants received a US $50 gift card for their participation in the study.

## Results

### Sociodemographic and Tobacco Use Data

Forty adults aged 21-69 years, with an average age of 43 (SD 13.6) years, participated in 8 focus groups (Table 1). Most participants were female, and the majority were non-Hispanic. Many participants endorsed current cigarette use. Fifty-five percent (n=22) reported use of electronic cigarettes or other vaping devices, while 40% (n=16) endorsed use of cigars.

**Table 1.** Demographics and tobacco use characteristics of participants (N=40).

Characteristics	Value
Age (years), mean (SD)	43 (13.6)
Sex (female), n (%)	23 (57)
Race, n (%)	
Black/African American	36 (90)
Black/African American and some other race, ethnicity, or origin	4 (10)
Ethnicity (non-Hispanic), n (%)	37 (92)
Sexual orientation, n (%)	
Straight	33 (82)
Gay or lesbian	2 (5)
Bisexual	5 (13)
Current cigarette use, n (%)	
Yes	35 (88)
Current other tobacco product use, n (%)	
Yes	31 (78)
Electronic cigarettes, e-cigarettes, or other vaping devices	22 (55)
Cigars, for example, little cigars, cigarillos, or large cigars	16 (40)
Smokeless tobacco, for example, chewing tobacco, snuff, or snus	6 (15)
Water-pipe tobacco or hookah	5 (13)
Other types of oral nicotine products, such as Velo lozenges, Rogue tablets, Lucy gum, or Pixotine toothpicks	1 (3)
Other	3 (8)
None of the above	9 (23)

Four central themes emerged about what mHealth app features were desirable by Black adults who use tobacco products. Each theme had 2‐3 subthemes that represented different domains of mHealth app features. See Table 2.

**Table 2.** Themes and subthemes with domains of mobile health app features represented.

Themes	Subthemes	Domain of app feature
Participants wanted representation and inclusivity through personalization and featuring people with similar lived experiences	Inclusion of images to represent the target population Personalization of the app Inclusion of content about relevant health risks	Representation and inclusivity User experience Content and inclusivity
Excessive targeting of the Black community was unappealing, and participants desired the app to feature a diversity of experiences rather than solely focusing on racial identity	Inclusion of testimonials from people with different backgrounds Outreach in the Black community	Content Social support
Participants desired accountability through trusted connections, and app tracking capability	Connection with health professionals Supportive anonymous community within the app App tracking capability	Professional support Social or peer support Tracking/user experience
Encouragement and motivation were more salient incentives than monetary rewards	Nonmonetary rewards Provision for diverse reward options	Gamification and rewards

### Theme 1: Participants Wanted Representation and Inclusivity Through Personalization and Featuring People With Similar Lived Experiences

#### Content and Inclusivity: Inclusion of Images to Represent the Target Population

When asked how to make the app more inclusive of Black adults, several participants emphasized the need to create an app that features Black adults.

*If we get on the app and we see a lot of us, then that will make us wanna use it more.*

[Focus group 1, Interviewee 1]

*You gonna get more people who’s like, “Hey, that applies to me. First of all, they look like me. They talk like me...They kinda think like me.”*

[Focus group 3, Interviewee 6]

One person lamented that it was not common to see themselves represented in existing apps.

*You know, like I need to see people of color, you know..., connecting my eyes because it’s not [in] a lot of apps out here...*

[Focus group 6, Interviewee 2]

Testimonials from fellow Black individuals were seen as a way to make people want to use the app:

...*having testimonials of other African-Americans, 'cause that would like make me at least feel more comfortable to even wanna try the app. Just seeing that other people that look like me have used it and have something positive to say.*

[Focus group 4, Interviewee 3]

#### User Experience: Personalization of the App

Some believed that inclusivity could be achieved through personalization of tobacco use and cessation goals.

*...[to] feel like you [are] talking to somebody that you know...maybe you could pick up from the personalized questions in beginning.*

[Focus group 6, Interviewee 2]

Participants also believed personalization could be achieved through in-app customization, which can allow people to have their goals at the forefront.

*...you should be able to customize* [the app], *you know, or personalize your experience with certain personal goals and..., based on your smoking habits...*

[Focus group 4, Interviewee 2]

*..Then this goal is set to be like the basis of the notification and the recommendations you receive.*

[Focus group 4, Interviewee 2]

#### Content and Inclusivity: Inclusion of Content About Relevant Health Risks

The inclusion of content about their health risks as Black adults who use tobacco products was also suggested:

*Include like the health aspect of it, like what smoking contributes to, like, just to add the statistics like, you know, African Americans,...make sure that you mention more of how it affects us as-as a culture.*

[Focus group 7, Interviewee 5]

With personalized content, participants hope to find stories to relate to.

*Personalize it because everyone has their own story. So, if you can find the testimonials that can represent you, that would work.*

[Focus group 1, Interviewee 2]

Some indicated that having personalized content would be preferable because they have different experiences and different stressors.

*...involve Black people [36], we have shared experiences that only [we] can understand...Like a Black mother or Black woman—if I need help, she could like advise me and pull me through.*

[Focus group 4, Interviewee 4]

*I think people that use cigarettes as a coping mechanism...I have—for example, stressors in the neighborhood...*

[Focus group 3, Interviewee 2]

### Theme 2: Excessive Targeting of the Black Community Was Unappealing, and Participants Desired the App to Feature a Diversity of Experiences Rather Than Solely Focusing on Racial Identity

#### Content: Inclusion of Testimonials From People With Different Backgrounds

While many participants voiced that the app should offer representation and inclusivity where Black adults are represented, many were adamant that the app should not focus on Black adults solely.

*I don’t wanna feel like it’s directed right at me ’cause I’m Black. ’Cause I’m more than just Black.*

[Focus group 2, Interviewee 2]

Rather, participants voiced that they wanted an inclusive app open to everyone struggling with smoking.

*It should be point to everyone with smokes, despite religion, despite your-your race, despite whoever you are.*

[Focus group 1, Interviewee 3]

*...provide practical solutions accessible to users from various income levels. You know, regardless of-regardless of whether you Black or not.*

[Focus group 4, Interviewee 2]

Participants suggested that one way to bring diversity into the app is to include testimonials from people from different backgrounds.

*I would like to hear,...different people’s testimonials...because we all have different stories, you know, and different reasons for doing the things that we do.*

[Focus group 1, Interviewee 1]

Having the app feature real stories was viewed as a way to motivate app use:

*...if you share like a real story of somebody that, you know, suffered bad consequences for smoking for not so long, that will probably motivate me to use the app.*

[Focus group 7, Interviewee 7]

#### Social Support: Outreach in the Black Community

Finally, some participants suggested conducting outreach at events within the Black community to promote the app and collaborate with respected figures:

*...have it displayed in a way that’s attractive to us, and then also present it in the areas that we are...events that we go to.*

[Focus group 6, Interviewee 4]

*You need to get...somebody famous. I’mma just use Diana Ross just for name sake, right? Say she used to smoke. You get her to talk about it. We all know, in the African American community, word of mouth is the most powerful thing we have.*

[Focus group 3, Interviewee 6]

Some participants suggested social media marketing:

*...if I have like a good TikTok video of someone that’s like a Black that’s...reviewing or giving me something to pull me in, that’s definitely gonna make me go and download it.*

[Focus group 6, Interviewee 4]

Another participant reflected on their experience of trying an app because it was recommended by an influencer:

*I actually got to know about it by...[a] Black female YouTuber and that’s how I got interested...*

[Focus group 4, Interviewee 4]

### Theme 3: Participants Desired Accountability Through Trusted Connections and App Tracking Capability

#### Professional Support: Connection With Health Professionals

When asked whether the app should offer connections to other app users, doctors, or other health care providers, participants expressed their desire to connect in order to promote accountability. Some participants indicated talking to professionals would help keep them accountable to their goals, either by providing advice, “that would seem pretty nice to be able to...have a coach that, you know, that you can go to for advice or assistance...” [Focus group 1, Interviewee 1] or by monitoring their progress, *“*I think you should have some kind of feature that to share your usage with your physician and stuff like that or your coach*”* [Focus group 4, Interviewee 2].

#### Social Support: Supportive Anonymous Community Within the App

When asked about connections with other users of the app, most participants indicated interest in seeking supportive accountability within the app:

*I think it will be better to have someone that, you know—‘cause my friend doesn’t smoke, so someone that understands. But to be able to hold you accountable.*

[Focus group 1, Interviewee 1]

Participants expressed a desire to decrease loneliness by connecting with others who understand what they are going through:

*I find that quitting smoking is like the loneliest thing I’ve ever dealt with in my life...[If] there’s like a little message board type of thing inside the app where people can just write in,...what they’re going through.*

[Focus group 1, Interviewee 2]

Most participants preferred the community within the app to be anonymous and were interested in anonymous interactions through groups, games, and a leaderboard, *“*to kind of promote data security...the information of the user should be de-identified or...be on the app as anonymous user” [Focus group 4, Interviewee 2] and “...in terms of being able to see what might be called like a leaderboard or a scoreboard...it can kind of make it...a bit more interactive” [Focus group 7, Interviewee 3].

Although most participants wanted a quit buddy within the app, they did not want this quit buddy to be a family member or friend. A few people noted that friends and family can be triggers for smoking and they are not understanding, “because sometimes family and friends can be triggers to smoking” [Focus group 8, Interviewee 3] and “sometimes like she said, you can’t really talk to family or friends about it, ‘cause they don’t—they don’t do it ‘cause they don’t understand it...” [Focus group 1, Interviewee 1]. While most participants did not want their friends and family connected to the app, some expressed that it would be helpful to have them understand and provide accountability: “It’ll give them an insight of what we’re going through” [Focus group 8, Interviewee 1] and “I think it’s a good idea to have, help because it could help you track your health and with how many smoking occurrences have you smoked per smoke a day, and can get your family involved and friends” [Focus group 8, Interviewee 7]. When asked if they wanted the app to connect with social media, some participants expressed fear of judgment and mean comments from other social network apps.

*You know, there’s some nasty comments, tellin’ you to just get over it.*

[Focus group 5, Interviewee 6]

*I don’t want that stuff flashed across Facebook and-and everybody to know what I’m tryin’ to do or attemptin’ to do because there’s a lotta unnecessary opinions.*

[Focus group 5, Interviewee 6]

#### Tracking and User Experience: App Tracking Capability

When asked about app tracking capabilities, most participants noted that they want to be able to track their usage to keep them accountable.

*Say, I might smoke this many a day. How ’bout the next day—and-and-and a tracker that helps me keep accountability.*

[Focus group 5, Interviewee 6]

A few participants noted that tracking their triggers through the app would allow them to learn what to avoid.

*It help you identify your triggers, and you’ll see,...what things cause you to smoke more so you could, like, lean away from it or stay from it, dependin’ on what it is. So, I think it’s [an] excellent idea.*

[Focus group 3, Interviewee 6]

Some participants expressed a desire to have frequent notification reminders to support accountability.

*So I need an app that’s going to be a constant reminder, almost an annoyance, you know, and - and to help me.*

[Focus group 8, Interviewee 2]

Most participants indicated that accountability could be supported through reminders of their goals.

*The daily goals are there, the notifications are there to remind you of your goals and all, so, uh, that should be helpful.*

[Focus group 4, Interviewee 5]

### Theme 4: Encouragement and Motivation Were More Salient Incentives Than Monetary Rewards

#### Gamification and Rewards: Nonmonetary Rewards

When asked about rewards, most participants expressed that encouragement and nonmonetary rewards were motivating.

*If you reach a goal, maybe,...you will get in this, a motivating statement, you know, as an incentive or a bunch of balloons goin’, confetti exploding, you could maybe get a star.*

[Focus group 2, Interviewee 1]

*Maybe even just a motivational somethin’, say, some kind of quote...even if it was just, “Good job. You - you did not smoke X number of hours today,” or “You smoked less by X percent today.” That’s the reward right there.*

[Focus group 8, Interviewee 3]

A few participants noted that receiving advice or feeling accomplished was rewarding.

*I just like when [it] lets me know that I’ve accomplished my goals for today.*

[Focus group 7, Interviewee 2]

*I just need somethin’ to keep me motivated, like a tip a day.*

[Focus group 5, Interviewee 2]

As one participant surmised, the app should be a place, *“*where you can get encouragement...or you can encourage somebody*” *[Focus group 2, Interviewee 1].

A few participants expressed that tangible incentives are not as encouraging as the benefits of quitting for one’s own health.

*Yeah, it’s not about the money, the incentives. It’s what I got to do to live longer.*

[Focus group 5, Interviewee 2]

*I have a little girl that I gotta live for, so I’m tryin’ to do better with- the smokin’...So that’s...motivating me to stop.*

[Focus group 6, Interviewee 7]

One participant noted that interacting with others within the app and reaching improvement milestones would be rewarding.

*I don’t really care to be rewarded, because I want to quit smoking. So, I just want the motivation there in the app, tracking...how many cigarettes that I, you know, didn’t smoke or whatever, you know, form of tobacco you use. And then just-just having the motivation...*

[Focus group 7, Interviewee 8]

#### Gamification and Rewards: Provision for Diverse Reward Options

Other participants expressed that although money is not the most important motivation, it may be important to some people; hence, allowing participants to choose their incentives was ideal. Some participants mentioned financial rewards, such as gift cards or payments.

*Some apps give you like daily points if you log in daily. So, if you can get a reward for logging in daily and then it could add up to getting the gift card, getting, you know, PayPal transfers, I think that would be pretty enticing.*

[Focus group 7, Interviewee 7]

*It always works better when there’s options versus you only have maybe one thing that you can do. So, it might be, oh, a cash app payout or Amazon, but then for the next person they might not really want that, they want something else.*

[Focus group 7, Interviewee 4]

## Discussion

### Principal Findings

As more mHealth apps for tobacco cessation are introduced each year, it is critical that these apps are built to serve everyone, including priority populations. Increasingly, apps tailored for Black adults have been introduced to confront health inequities, with an emphasis on iterative design using formative work from members of the target population [37]. Themes and subthemes from the focus groups revealed a strong preference for certain features, including the type and presentation of content in the app, inclusivity, user experience, tracking capability, professional and social support, gamification, and rewards. Preferred features also aligned with components of the self-determination theory, thereby enhancing the significant role that these features may play in the usage of an mHealth app. These findings provide insight into what app features deter or promote app receptivity, and how to attract Black adults who use tobacco products to use an mHealth app for smoking cessation.

### Comparison With Previous Work

In our study, we found that participants desired an app with features that highlight representation and promote inclusivity. To do this, participants recommended that an mHealth app should include testimonials, images, and videos featuring Black adults. Prior studies examining preferred features of mHealth apps for Black women have found that participants desire an app to include a directory of nearby Black female health care providers within their community [38,39]. Having racial concordance with a health care provider can help participants feel they have someone with whom they can relate [39]. One way that representation can be achieved in an mHealth app for tobacco cessation is to have a video testimonial by a Black former tobacco user included in the app, with the person sharing their experience with quitting as well as the health benefits experienced after quitting.

We also found participants desired in-app personalization of content and user experience based on their attributes, such as race, gender, and tobacco use habits and experience. Our findings suggest that to improve the user experience, mHealth apps should allow for personalization based on several factors, not just race. This may lead to a more robust experience for the app user. Further, several participants emphasized how the hypothetical mHealth app should feature a diverse range of people with different perspectives, experiences, and identities. This is consistent with findings in one study on app development where participants desired a diversity of people within an app, specifically wanting “everyone” to be included [40].

Similar to our study, previous research has shown that people are interested in an app with relevant health information and statistics [28]. This finding suggests that when health risks and statistics are personalized, the data are more relatable. Providing personalized content and feedback is associated with increased engagement with mobile app interventions [41]. For example, in an mHealth app for tobacco cessation, including statistics about the rate of cardiovascular disease or cancer for Black tobacco users compared with nontobacco users may increase engagement.

Another important finding in this study is that while participants were in favor of representation within the app, they were understandably hesitant or resistant to an app that focused solely on their racial identity. A previous study on mHealth apps for smoking cessation noted similar concern [28]. This perspective may stem from the justified mistrust of the health community and historical mistreatment of Black people in health care and research [42-44]. In a study that examined mHealth research receptivity among Black men, over a quarter (27%) of men and nearly a third of Black women (30%) identified “mistrust of researchers” as a barrier to participation [45,46].

While a more inclusive but nontargeted app is preferred, outreach strategies to reach the Black community were suggested, including marketing at events for the Black community, marketing by Black influential figures or celebrities on social media, and promotion by Black influencers. This finding suggests that having a known source may engender feelings of trust [47]. Furthermore, in recent years, health campaigns have begun working with influential figures such as pastors or influencers to promote health interventions, including vaccine campaigns [47] and physical activity and healthy eating [48,49]. Influencers can leverage their social influence and reach their audiences directly to promote health interventions [50,51], which could be used to increase app receptivity within the Black community.

In addition, with the high rate of social media use by Black adults [25] and the inclusion of social media as a tool for health promotion and intervention dissemination due to wider reach, scalability, and cost-effectiveness [52], targeted social media campaigns can be beneficial. In tobacco cessation studies, the use of social media platforms for recruitment has shown success, especially for minority populations that are typically hard to reach [49]. In one study by Bricker et al [52], tailoring of a social media campaign to disseminate an mHealth app in a way that focused on self-control and health values was found to be cost-effective and associated with high reach based on the number of clicks and conversion to app installation. The use of social media platforms to disseminate an mHealth app for tobacco cessation targeted for Black adults can increase the likelihood of awareness of the mHealth app, which may convert into more interest and greater use.

Furthermore, prior research to investigate smokers’ reactions on social media platforms revealed that comments and the type of reaction such as “love” or “haha” were a reflection of the level of motivation with regard to readiness to quit [53]. This suggests that although social media can be used to enhance the visibility of mHealth apps for tobacco cessation, it can also stimulate motivational processes by serving as a space for cessation-related posts that contain culturally representative narratives, prompting users to share their own motivations and goals for quitting or positively respond to comments that are reflective of their own experiences. This way, social media is not only a tool to promote the mHealth app but can also help to assess qualitative measures such as levels of engagement and motivation to quit.

Our findings also revealed the importance of an in-app community that offers accountability through supportive connections (eg, through health care providers and quit buddy programs). Previous research on mHealth apps has found that many people desire the ability to connect with health care providers within apps [39]. One study examining supportive accountability through counselor monitoring and supportive advice found that having a trusted expert within an mHealth app intervention for smoking cessation led to increased app engagement [54]. In another intervention that used peer-to-peer exchanges between participants seeking smoking cessation resources and communications from experts with treatment-related questions for participants to discuss, doubled sustained abstinence was observed compared to those who only used nicotine patches and had access to a cessation website [55]. Further, the effectiveness of mHealth interventions can be enhanced by human and social support [56,57].

Although open to connections, participants were hesitant and less receptive to having family or social media integrated within the app due to privacy concerns. Other studies examining receptivity to social media on mHealth apps have similarly found that most participants did not want social media integration, citing privacy concerns [39]. Although privacy is a major concern, social media play a significant role by serving as a platform through which health-related behavior change interventions can be delivered and health-related connections or support groups developed in a meaningful way [58,59]. For Black adults who use tobacco products, addressing the privacy concern in a transparent way may improve receptivity to the integration of elements of social media into the intervention.

In contrast, some people prefer having emotional support, such as encouragement, from friends and family [60,61]. Studies have found that stigma against individuals who smoke can prevent them from seeking care [62]. One study found that negative comments from one’s social network about their smoking habits can even lead to a decrease in quitting progress [61]. To combat this concern, any external connections to the app should be optional.

App tracking capability was also an important feature for accountability and has been identified as a desirable feature and method to promote accountability in other studies [39,54]. Several mHealth interventions use the tracking feature to help monitor chronic health conditions and medication intake [63,64]. One qualitative study that explored experiences of Black adults who use tobacco products with a specific mHealth app for smoking cessation identified that the tracking feature was highly preferred [28]. With mHealth apps for tobacco cessation, tracking of the rate of use of tobacco products, cravings, or money saved over a period of time from less use can lead to sustained motivation and cessation-related behavior.

Lastly, we found that while some participants said that financial incentives could attract some people to use the app, many participants did not think financial incentives were necessary to attract users to the app. Prior research suggests that financial incentives can modify or increase adherence to desired behavior changes, like exercise [65], dietary changes [66], or engagement with a state Quitline [67]. However, the sustained provision of financial incentives may not be feasible for mHealth apps long term, nor are they always necessary. In one study examining motivations and barriers to participating in mHealth research among Black males, approximately 40% indicated that offering financial incentives would motivate participation, while only 16% indicated that a lack of financial incentives was a barrier to participation [45]. Similar rates were also seen among Black women, 39% and 15%, respectively [46]. Continued engagement with an app is further augmented by other considerations, like the content shared [68]. Therefore, while financial incentives are desirable, their absence may not significantly deter participation with mHealth interventions. This finding aligns with existing research that the gamification (eg, inclusion of game elements and rewards) of mobile apps can reward and motivate app users [69,70] and promote behavior change [71].

### Design Implications for App Development

To enhance the translation and application of our findings to an mHealth app development, we mapped out each subtheme and domain feature into examples of concrete mHealth app features with pertinent processes from the self-determination theory. We also included examples of ethical and privacy considerations, as well as potential metrics to consider during implementation. The map, as outlined in Table 3, shows how representation and inclusivity, personalization, content, accountability, tracking and user experience, social and professional support, and nonmonetary incentives can be operationalized into actual, concrete features within an mHealth app for tobacco cessation.

**Table 3.** Design implications for mobile health app with preferred features.

Subthemes and domains of app features	Examples of concrete app features	Self-determination theory components	Privacy/ethical considerations	Potential metrics
Representation and inclusivity	Video testimonials from other Black tobacco users Culturally relevant language	Relatedness Autonomy	Avoidance of identifying information of individuals in videos or photos Opt-in for culturally relevant material, instead of it being the default Informed consent Avoidance of racial profiling in content	Amount of time spent and frequency of viewing culturally relevant information Rate and frequency of opt-in for culturally relevant material
Personalization	In-app customization of goals, quit date, reminders, and prompts Personalized diary	Autonomy	User-controlled input of information Password-protected storage and opt-out option	Achievement of personal goals Number and frequency of diary entries
Content	Health risk statistics relevant to the target user	Relatedness Competence	Ensure credibility of the source of information Avoidance of the use of sensitive language or medical jargon	Completion of content sections
Accountability	Quit buddy, daily check-in	Autonomy Relatedness	User opt-in Anonymization of profile	Daily active use
Tracking and user experience	Tracking tools for mood, cravings, number of cigarettes used, and days without use	Autonomy Competence	User control on the type of information to track or delete Secure or password-protected storage of tracked data	Daily active use
Social support	Anonymous in-app community	Relatedness	Anonymization of user profile User opt-in for participation or for integration with social media	Number of users who opt-in Number and frequency of comments and posts on the community board
Professional support	Quitline or access to a tobacco treatment specialist, trusted expert chat	Competence	Informed consent to interact with a professional Encryption of messages	Number and frequency of use or messages sent to an expert Number of times the Quitline button or icon is used
Nonmonetary incentives	Badges, points	Competence	Option for private mode where reward is only seen by the user No shaming or negative language during relapse	Rate of badges or points earned

### Limitations

A limitation in this study is that findings may not be generalizable. First, we used a convenience sample online and recruited from one social media platform. This may have limited the sample size as well as the diversity of participants in terms of gender, age, educational level, and income. Conducting the focus groups online, as well as the requirement of a history of app usage or willingness to use an mHealth app in the future for participation in this study, further limits the diversity of perspectives about features of mHealth apps for tobacco cessation. Furthermore, findings may also not be generalizable to Black adults outside of the United States due to cultural differences and other factors that may exist across countries. Obtaining the perception of Black adult tobacco users who are not receptive to the idea of mHealth apps for tobacco cessation may provide additional insight into how best to develop and market these apps in a more inclusive way.

### Conclusions

Our findings confirm the need to design mHealth apps for tobacco cessation with features that are reflective of cultural relevance, inclusivity, and diversity of experiences without excessive targeting and support motivation as stated in the self-determination theory [19-21]. Consistent with Perski and colleagues [72], engagement with mHealth apps increases when design features support personal relevance, motivation to quit, and credibility. Our findings provide insight into the improvement of existing apps and future development of an mHealth app that can appeal to Black adults who use tobacco products, potentially increasing app use, engagement, and successful cessation. Findings from this research can also serve as a groundwork for future research to explore the effectiveness of an mHealth app culturally tailored with preferred features optimized to enhance engagement by Black adults who use tobacco products and to identify other factors that may improve usage and lead to better outcomes for the target population.

## Supplementary material

10.2196/63340 Multimedia Appendix 1 Focus group guide.

## Abbreviations

mHealth: mobile health
