# Supplementary material for: Features of Mobile Health Apps for Tobacco Cessation That Appeal to Black Adults Who Use Tobacco Products: Focus Group Study
Source: JMIR Mhealth Uhealth. 2026 Feb 6;14:e63340. doi: 10.2196/63340 (PMC12880593; doi:10.2196/63340)
Supplement: Multimedia Appendix 1 [file mhealth-v14-e63340-s001.pdf]

Focus group guide

Note: This is a semi-structured focus group guide and the exact questions asked will vary based on the responses of participants.

Introduction:

Thank you for agreeing to participate in this focus group and share your thoughts about mobile health apps for smoking or tobacco use cessation and the different features or characteristics of these apps. Your participation is highly appreciated and will help us determine what features need to be included in a future app to help people stop smoking and/or using tobacco products. My name is [interviewer name] and I will be leading today's focus group. [Assistant's name] will be assisting and taking notes in the background.

The purpose of this research study is to determine which features of mobile health apps for smoking/tobacco use cessation are most desired by African American adults who currently smoke. African Americans have not been well represented in prior studies of mobile health apps for smoking/tobacco use cessation despite the fact that 14.6% of African Americans smoke at least on some days and African Americans have one of the highest rates of smoking-related health conditions in the U.S.

We want to identify features about mobile health apps that appeal to African American smokers. The goal is not to develop an app only for African American smokers but to develop one that is inclusive to African American individuals.

Today's focus group will last about an hour and you will receive a $50 gift card by email upon the completion of your participation in the study.

Please remember that you may choose to end your participation at any time, and you can skip any questions that you don't want to answer. Your responses will be compiled with all other participants' responses and no one's name will be included in any reports about the interviews.

This interview will be audio and video recorded. The recordings will be transcribed for analysis. To protect your identity as a research subject, the research data will not be stored with your name and the researchers will not share your information with anyone. All research data will be stored electronically on a university secure server in a password protected folder or secure drive (e.g., private Microsoft Teams page) which will only be accessible to the immediate study team requiring access to it. Using the recordings of focus groups, we will create transcripts. Recordings and resulting transcripts will be destroyed once data analysis is complete. In any publication about this research, participant identifiers (like name) will not be used. To help protect the privacy of every participants, we ask that you not to share any information you may hear during the focus group with others. Thank you!

- Do you have any questions before we start? Is it ok to begin the interview?
    - Is it ok if we start the recording now?
        - **[If yes]:** Say "I will begin recording now."
        - **[If not ok with recording interview]:**

- Ask “why not?” [since they agreed to this in the consent]
- [Continue with the interview and take notes, but we may not be able to use responses in the analysis]

Smoking/Tobacco-Use Cessation History:

1) You already answered some questions about your smoking/tobacco use history in the survey, but we would like to get a better understanding of your experience. Can you briefly tell me a bit about your history with tobacco products?
   a. Probes
      i. What tobacco products do you use and when did you start using them?
      ii. Have you attempted to quit smoking/tobacco products?
         1. [If yes]:
            a. Tell me about your experience with quitting. Were you successful?
         2. [If no]:
            a. What are some reasons you have not attempted to quit?
      iii. Did anything change about your tobacco use since you completed the survey?

Experiences with Apps:

Now we would like to ask you about your history of mobile health app use. By apps, we mean programs that you download from the internet and use on mobile devices like smartphones or tablets.

1) Have you ever used a mobile health app on your phone, tablet, or handheld device to monitor your health or lifestyle choices? For example, MyFitnessPal?
   a. **[If have used an app]:**
      i. What made you download the app?
      ii. What did you like about the app?
      iii. What did you dislike about the app?
      iv. What features do you wish the app had?

   b. **[If they have not used an app]:**
      i. What has prevented you from using a mobile health app?
      ii. What would make you interested in using a mobile health app?

2) Have you ever used an app to help you quit smoking or using tobacco products?
   a. **[If have used an app]:**
      i. Which app(s) did you use?
      ii. What was your experience using this app (these apps)?
         1. **[If used multiple apps]:** Which apps were most helpful? Why?
         2. What features of the app(s) were helpful?
         3. Which features do you wish you could have changed?

        4. Were you able to successfully quit a tobacco product using an app? If yes/no, why do you think you were/were not successful?
        5. Would you be willing to use the same app again?
  b. **[If haven't used an app]:**
    i. Are there particular reasons why you have not used an app to help you quit smoking or using tobacco products? If so, what are they?
    ii. What has been preventing you from trying out an app?
        1. [Move to next section asking about experience with other apps]
    iii. What barriers have you experienced when using an app?
        1. What do you dislike about apps?
    iv. Would you be interested in using an app to help you quit smoking or using tobacco products in the future? Why or why not?

Today we will discuss features of different mobile health apps for smoking and tobacco use cessation. We are mainly interested in your thoughts about different features that make up mobile health apps for smoking and tobacco use cessation.
Although we have made a list of features to guide us, feel free to bring up any features that you found helpful or not helpful.

Preferred App Features/Designs:
We are hoping to use feedback from these interviews to help us design an app that is inclusive to African American individuals to help people quit. We'd like to ask for your opinion on some specific features we might include in an app.

(See list of features and levels that will be used for the focus group discussion)

1. [Content] What type of information do you think would you want an app to have and would motivate you to use the app?
  a. Probes:
    i. **Benefits of Quitting**: Health benefits, Financial benefits, Other physical benefits, Consequences of smoking vs benefit of quitting (positive vs negative framing), Inclusion of health calculators
    ii. **Strategies for Quitting**: Info about other smoking cessation methods (e.g., NRT), Info to develop behavioral strategies for smoking cessation, Inclusion of distractions, Alternatives for coping with stress, Identify personal value-based inspiration for quitting
    iii. **Quit Plan:** Personalized vs General plan, Dealing with relapse, Dealing with withdrawal symptoms

2. [User Experience] What type of interactions do you want to have with the app?
  a. Probes:
    i. Website links to other cessation resources or programs
    ii. Inclusion of health calculators
    iii. Active learning e.g. gamifying
    iv. Short videos/Inspirational stories for motivation

v. Positive reinforcement/reward
vi. Information entry methods
vii. Pop-up notification/reminders

b. How frequently would you want to receive notifications from the app?
c. What type of images would you like to see?
d. Would rewards (e.g., badges, stars) for quitting behaviors be motivating? Would the use of images related to harms of smoking vs benefit of quiting,

3. [Functionality: Personalization] What personalization options would be helpful to have in the app?
   a. Probes:
      i. Ability to customize notification messages
      ii. Set own quit date
      iii. Tailored feedback in response to information entered
      iv. Personalization of your: reason for quitting, uploading of visuals for encouragement, motivational song or video
      v. Ability to filter smoking cessation tips based on situation e.g. boredom vs stress
      vi. Ability to create profile/upload photo/preferred music/videos about reasons for quitting

4. [Functionality: Tracking] What type of tracking would be helpful for the app to include? (E.g., tracking urges, products used, progress).
   a. Probes:
      i. Tracking of number of cigarettes smoked per day or number of days without smoking.
      ii. Tracking of urges, cravings, smoking location, triggers, mood and physical symptoms
      iii. Tracking number of times quitting tips were applied
      iv. Keeping a journal in the app
5. [Privacy & Security] What privacy and/or data protections would you want to see in the app? (E.g., tracking urges, products used, progress).
   a. Probes:
      i. GPS or location tracking
      ii. Need for wifi or internet connection for app usage
      iii. Maintains privacy of user information
      iv. Stores information on the phone vs secure cloud storage
      v. Ability for user to control privacy/sharing
      vi. Ability to block other app users
      vii. Options to turn off GPS tracking
6. [Social Network] Should we integrate your social network into the app interface? If yes, how?
   a. Probes:
      i. Messages from family or friends
      ii. Ability to share information from app with loved ones
      iii. Connection with other users of the app
      iv. Quit buddy system
      v. Support groups

vi. Addition of friends to the app
vii. Report progress towards quitting by email or social media
viii. Social interactions that involved sharing of tips, responses such as likes, favorites for each distraction

Should the app have connections to other app users, doctors, or other healthcare providers (e.g., quit coach?)

b. Probes
   i. Communication with other experts, with healthcare team
   ii. Connection with a quit coach or sponsor

7. [Inclusivity] Earlier, I mentioned how African Americans have not been well represented in prior studies of mobile health apps for smoking/tobacco use cessation. How can we make apps more inclusive and desirable for African American individuals?
   a. Probes:
      i. Images of Black people or those of other minority groups
      ii. Health information particular to Black individuals as it relates to smoking
      iii. Inclusion of success stories from other Black individuals
      iv. Information about cigarettes brands commonly used by Black smokers e.g. Newport
      v. Information about targeted marketing in Black communities
      vi. Information about substances added to cigarettes which them highly addictive e.g. menthol
      vii. Culturally relatable music
      viii. Race based content vs neutral
8. Other features (including those that may come up in the discussion)
9. What would your ideal app for tobacco cessation look like?
   a. Probes:
      i. If you could wave a magic wand and create the perfect app, what would it look like?
      ii. Would it include tracking smoking/tobacco use behavior? For example, recording when you have the urge to smoke, events going on that trigger the urge, and whether you were able to fight the urge to smoke or not?
      iii. Would it include messages from family or friends to remind you not to smoke?
      iv. Would it include a way to connect with others using the app such as Facebook or WhatsApp groups [social networking]?
   b. Look & feel

10. Is there anything else we should keep in mind when designing an app to help people quit smoking or tobacco use so that it is inclusive to African American individuals? This could be informed by your experience with other health applications.

Closing Statement:

"Those are all the questions I wanted to ask you today. Thank you again for your participation in this study. You will receive your electronic gift card by email within the next 10 business days."
